# Profiling Mealtime Verbal Interactions between Nursing Home Staff and Persons with Dementia

**DOI:** 10.1093/geroni/igaf122.3645

**Published:** 2025-12-31

**Authors:** Annika Ellis, Kyuri Lee, Wen Liu

**Affiliations:** University of Iowa, Eagan, Minnesota, United States; The University of Iowa, Iowa City, Iowa, United States; Penn State University, University Park, Pennsylvania, United States

## Abstract

Effective communication is crucial to Person-Centered Care (PCC) for Persons with Dementia (PWD) during mealtimes as verbal interactions can influence engagement. This study analyzed 12,959 verbal behaviors in 245 full-mealtime videos in long-term care settings using the CUED coding scheme. Staff produced 9,176 positive verbalizations, including “Orientation/Giving Instruction” (24.80%) and “Giving Choices” (16.46%). Of staffs’ 1,781 negative verbalizations, most involved side conversations (42.57%). Residents contributed 2,066 verbalizations in the dyadic conversations (86% positive, 14% negative). Notable resident unintelligible utterances were coded as “Unsure – positive” (30.66%). Additional common verbal resident behaviors included “Expressing personal need or preference” (16.33%) and “Showing approval/agreement” (16.28%). Together, these dyadic interactions indicate an overall positive conversational tone with relatively infrequent negative themes, facilitating a deeper understanding of the context within dementia care communication. Pearson correlations revealed significant associations between staff and resident behaviors. Staff positive behaviors were positively correlated with both resident positive (r = .288, p < .001) and negative behaviors (r = .275, p < .001), suggesting that staff engagement prompted a range of resident responses. Staff negative behaviors were associated with resident negative behaviors (r = .153, p = .016). Additionally, longer durations of staff’s negative side conversations were correlated to increased number of total negative staff behaviors (r = .173, p = .007). These associations indicate that while staff demonstrate many PCC behaviors, negative or off-topic conversations can still shape resident responses in unhelpful ways. Strengthening staff communication strategies to remain focused, positive, and inclusive may enhance mealtime engagement for PWD.

